# Enhanced Response of ZnO Nanorod-Based Flexible MEAs for Recording Ischemia-Induced Neural Activity in Acute Brain Slices

**DOI:** 10.3390/nano15151173

**Published:** 2025-07-30

**Authors:** José Ignacio Del Río De Vicente, Valeria Marchetti, Ivano Lucarini, Elena Palmieri, Davide Polese, Luca Montaina, Francesco Maita, Jan Kriska, Jana Tureckova, Miroslava Anderova, Luca Maiolo

**Affiliations:** 1 Consiglio Nazionale Delle Ricerche, Istituto per la Microelettronica e Microsistemi, 00133 Rome, Italy; ignacio.delrio@artov.imm.cnr.it (J.I.D.R.D.V.); ivano.lucarini@cnr.it (I.L.); elena.palmieri@artov.imm.cnr.it (E.P.); davide.polese@cnr.it (D.P.); luca.montaina@artov.imm.cnr.it (L.M.); luca.maiolo@cnr.it (L.M.); 2 Alma Mater Studiorum—Department of Engineering, Università di Bologna, 40126 Bologna, Italy; 3 Institute of Experimental Medicine, Czech Academy of Sciences, 1083 Videnska, 142 20 Prague, Czech Republic; valeria.marchetti@iem.cas.cz (V.M.); jana.tureckova@iem.cas.cz (J.T.); miroslava.anderova@iem.cas.cz (M.A.); 4 Second Faculty of Medicine, Charles University, 84 V Uvalu, 150 06 Prague, Czech Republic

**Keywords:** acute brain slices, cerebral ischemia, micro/nano electrode array, spreading depolarization, zinc oxide nanorods

## Abstract

Brain ischemia is a severe condition caused by reduced cerebral blood flow, leading to the disruption of ion gradients in brain tissue. This imbalance triggers spreading depolarizations, which are waves of neuronal and glial depolarization propagating through the gray matter. Microelectrode arrays (MEAs) are essential for real-time monitoring of these electrophysiological processes both in vivo and in vitro, but their sensitivity and signal quality are critical for accurate detection of extracellular brain activity. In this study, we evaluate the performance of a flexible microelectrode array based on gold-coated zinc oxide nanorods (ZnO NRs), referred to as nano-fMEA, specifically for high-fidelity electrophysiological recording under pathological conditions. Acute mouse brain slices were tested under two ischemic models: oxygen–glucose deprivation (OGD) and hyperkalemia. The nano-fMEA demonstrated significant improvements in event detection rates and in capturing subtle fluctuations in neural signals compared to flat fMEAs. This enhanced performance is primarily attributed to an optimized electrode–tissue interface that reduces impedance and improves charge transfer. These features enabled the nano-fMEA to detect weak or transient electrophysiological events more effectively, making it a valuable platform for investigating neural dynamics during metabolic stress. Overall, the results underscore the promise of ZnO NRs in advancing electrophysiological tools for neuroscience research.

## 1. Introduction

Neural interfaces have undergone significant advancements in recent years, with flexible microelectrode arrays (fMEAs) emerging as a powerful tool for high-resolution electrophysiological recordings [1]. Unlike traditional rigid electrodes, flexible MEAs conform to biological tissues, minimizing mechanical mismatch and improving long-term stability [2,3]. The stiffness of brain tissue (~100 Pa–10 kPa) contrasts significantly with polymeric implants, which range from 1 GPa (polyimide-based) to 1 MPa (PDMS-based) [4,5]. The use of ultra-thin fMEAs (less than 5 µm in thickness) mitigates this mismatch, minimizing the foreign body response. Recent studies have also explored laser-induced graphene systems, conductive hydrogels, and advanced bioelectronic platforms to further enhance the multifunctionality, biocompatibility, and integration of flexible neural interfaces [6,7,8]. Furthermore, the use of nanostructures enhances electrode performance by increasing the electrode’s surface area, thus improving its impedance. Additionally, different nanostructures exhibit peculiar properties in terms of biocompatibility, cell differentiation, cell coupling, etc., and they interact differently with the cells depending on their shape, distribution (ordered and aligned or disordered structures), and surface functionalization. The effect of disordered nanostructures on neuronal and glial activity has been widely explored using organic fibers such as PEDOT-based scaffolds and carbon nanotubes (CNTs), which have demonstrated excellent electrical conductivity and biocompatibility for neural interfaces [5,9]. Recent studies have also advanced the field through organic nanostructured interfaces supporting enhanced neural integration [10,11]. Among the diverse materials, zinc oxide nanowires (ZnO NWs) [12,13,14] and nanorods (ZnO NRs) [15,16] show interesting features for biointerface applications. Indeed, nanostructured ZnO is well-known for the wide variety of nanostructures that can be grown using scalable and low-temperature methods, for its remarkable optical and electrical properties (such as the possibility to tune the material’s resistivity over several orders of magnitude), and for its potential piezoelectricity [17,18,19]. Compared to these organic materials, ZnO nanorods offer high mechanical stability and piezoelectric properties, yet their crystallinity and surface reactivity might pose challenges for long-term biointegration. In contrast, Si nanowires provide a more established surface functionalization chemistry and efficient photothermal generation due to enhanced light trapping, but lack the inherent transparent optical feature of ZnO NRs [20] and their ability to grow directly on the device substrate due to the low temperature of synthesis of ZnO nanostructures (around 90 °C). Growing Si nanowires directly on low glass transition temperature flexible substrates like polyimide is challenging since catalytic Si NW growth is triggered at 320 °C (a temperature close to the glass transition temperature of polyimide), producing low-density, disordered mats compared to ZnO NRs. Therefore, the choice between ZnO nanorods and Si nanowires depends on balancing biointerface stability, mechanical resilience, and desired electrophysiological performance. In this study, disordered ZnO nanorods were intentionally selected over ordered arrays due to their simpler and more scalable fabrication, as well as their ability to increase the effective surface contact area with neural tissue, offering multiple anchoring points for cells and facilitating cell engulfing. This random orientation can promote a more intimate and conformal electrode–tissue interface, improving charge transfer while avoiding complex lithographic patterning steps. These advantages make disordered ZnO nanorods a practical and robust choice for flexible neuroelectronic applications [21].

In the case of brain interface and electrode fabrication, ZnO NRs can be coated with different metals (Au, Ti, Pt, etc. [14]) or combined with 2D materials [22,23,24] to control the device impedance and tune the electrode properties in terms of signal recording and/or brain stimulation tasks. Nanostructured flexible interfaces have already been reported as valuable electrophysiological tools to investigate brain activity in both healthy and pathological conditions [25,26], with the aim of identifying peculiar signal patterning and unraveling cognitive processes. In particular, some disordered nanostructures have been implemented not only for neurons but also as glial platforms to monitor, for example, the ion current exchange among astrocytes during the different physiological mechanisms [27]. In this respect, the usage of nanostructured MEAs provides additional information in electrophysiological measurements, thus opening up interesting insights into the interpretation of these bio-signals.

Flexible microelectrode arrays (MEAs) offer substantial advantages for real-time neural recording thanks to their conformability, biocompatibility, and ability to reduce tissue damage compared to rigid systems. Their mechanical compliance allows stable, long-term recordings both in ex vivo models and during chronic in vivo experiments. The integration of nanostructured materials with flexible MEAs further enhances their performance, improving electrode–tissue coupling, reducing impedance, and increasing sensitivity to subtle neural signals. This synergy makes flexible nanostructured MEAs particularly well suited for monitoring fine-scale neural dynamics under complex and evolving pathological conditions.

One key application of these advanced platforms is the study of ischemia, a condition defined by reduced cerebral blood flow that deprives brain tissue of oxygen and glucose, leading to ATP depletion. This metabolic collapse triggers a cascade of cellular responses affecting both neurons and glial cells. A hallmark of ischemic pathology is the disruption of ionic homeostasis, severely compromising neurons and astrocytes. The failure of key ATP-dependent ion pumps, such as Na^+^/K^+^-ATPase and Ca^2+^-ATPase, results in intracellular Na^+^ accumulation, elevated extracellular K^+^, and increased Ca^2+^ influx [28]. Consequently, neurons undergo rapid and sustained depolarization, triggering excessive glutamate release and promoting excitotoxicity [29]. This ionic dysregulation further propagates into spreading depolarizations (SDs), waves of near-complete depolarization of neurons and glia across the gray matter. Under ischemic conditions, recurrent SDs exacerbate ATP depletion and ionic imbalances, promote further glutamate release, and worsen neuronal damage [30].

Astrocytes are essential regulators of ion and neurotransmitter homeostasis under both physiological and pathological conditions. They buffer extracellular K^+^ and uptake glutamate, maintaining neuronal excitability and protecting against excitotoxicity. However, under substantial ischemic stress, astrocytic function is severely impaired. Their diminished buffering capacity and disrupted glutamate uptake facilitate SD propagation, impair neuron–glia communication, and contribute to secondary injury through the release of pro-inflammatory cytokines [31]. Thus, brain ischemia induces profound ionic disturbances that drive neuronal depolarization and recurrent SDs. The progression of ischemic injury is tightly linked to the collapse of ion homeostasis, particularly within neurons and astrocytes.

These events can be effectively monitored using extracellular electrodes that detect changes in local field potentials, typically observed as negative shifts in the direct current (DC) potential, along with alterations in extracellular ion concentrations.

To explore these pathophysiological mechanisms in a controlled environment, acute brain slices are used as an ex vivo model, offering a well-preserved local circuitry and cellular microenvironment. This approach allows for precise manipulation and high-resolution recording of neural activity during ischemia-like conditions [32,33]. While brain slices maintain much of the intrinsic network architecture and cellular composition, they also present limitations, such as reduced neuronal density, disrupted long-range connectivity, and diminished spontaneous activity, when compared to in vivo conditions. Despite these constraints, acute slices remain a widely used model for investigating fundamental neurophysiological processes in a controlled setting, particularly when combined with sensitive recording platforms that enhance detection of sparse or low-amplitude neural signals.

In this study, we fabricated, characterized, and applied nanostructured fMEAs (nano-fMEAs) for recording ischemia-induced neural activity in acute brain slices. These nano-engineered electrodes were designed to enhance sensitivity and improve the signal-to-noise ratio under ischemic conditions. Here, we demonstrate their efficacy by recording neural responses under both OGD and hyperkalemic conditions, and by benchmarking them against conventional flat electrodes of equivalent dimensions. Our findings underscore the superior performance of nano-fMEAs compared to the flat flexible MEA in detecting ischemia-induced electrophysiological changes with higher fidelity. This platform offers a powerful tool for studying ischemic pathophysiology and evaluating potential neuroprotective strategies.

## 2. Materials and Methods

### 2.1. Fabrication of Flexible Microelectrode Arrays (fMEAs)

#### 2.1.1. Substrate Preparation

The fabrication of flexible microelectrode arrays (fMEAs) follows a multistep process, beginning with the preparation of a rigid substrate. A 3-inch silicon oxide (SiO_2_) wafer, used as a polyimide holder, is cleaned in a 5% hydrofluoric acid solution for 5 s to remove impurities, followed by rinsing with deionized water and drying under nitrogen flow. A 4 µm thick layer of polyimide PI2611 (HD Microsystems™ GmbH, Greifswald, Germany) is utilized as the substrate for the flexible microelectrode array [34]. The polyimide is applied to the wafer by a spin-coating process (2000 RPM for 60 s). The sample is then cured at 250 °C for 4 h in a nitrogen atmosphere. Following polyimide curing, reactive ion etching is performed under these conditions: 90% oxygen, 20% power, and 45 mTorr pressure for 30 s to increase polyimide surface roughness and improve metal adhesion or zinc oxide nano-precursor deposition. Two types of fMEAs are fabricated: standard flat fMEAs (flat fMEAs) and nanostructured fMEAs (nano-fMEAs).

#### 2.1.2. Zinc Oxide Nanorod Growth

For the nano-fMEAs, ZnO NRs were synthesized via chemical bath deposition. For this process, the wafer with the first polyimide layer is spin-coated with a 0.1 wt% solution of zinc oxide nanoparticles (PT Merck Chemicals and Life Sciences, Jakarta Timur, Indonesia), which act as seeds for the nanostructure growth. The growth occurs in a 2.1:1 zinc nitrate hexahydrate and hexamethylenetetramine (Zn(NO_3_)_2_:HMTA) aqueous solution at 85 °C for 60 min [24,35,36,37]. Afterwards, the sample is cleaned and dried, and the procedure continues as with the traditional fMEA.

#### 2.1.3. Photolithography and Metal Deposition

Once both flat fMEA and nano-fMEA substrates are prepared, a photolithography process is used to define the recording sites and connecting tracks [38]. Metal evaporation is used to deposit 25 nm of Ti to improve adhesion, followed by 125 nm Au to form a thin metal bilayer, and a lift-off procedure is adopted to define the metal layout. To passivate the device, an additional polyimide layer is deposited following the same procedure used for the substrate. Electrode areas are then exposed by wet etching to uncover the Au-coated ZnO nanorods (ZnO NRs). Finally, holes are patterned in the MEA grid using photolithography followed by reactive ion etching (RIE) with an O_2_/CF_4_ gas mixture at a working pressure of 50 mTorr and a plasma power of 90 W for 1 h. These perforations prevent tissue asphyxiation by ensuring adequate oxygenation and facilitating a more uniform distribution of nutrients to the tissue slice.

#### 2.1.4. Device Assembly and Connectivity

Each fMEA and nano-fMEA is mechanically detached from the wafer and bonded to a custom printed circuit board (PCB), which acts as an interface to connect the MEA to the recording board. The acquisition system, an in-house custom-made recording board, supports up to 32 channels and enables real-time data visualization through customized software written in MatLab (R2023a) [26]. This system can amplify, filter, and digitize the signals through an RHD2000 chip by INTAN Technologies (Los Angeles, CA, USA) The low-noise amplifier can reach a maximum value of 200×.

### 2.2. Device Characterization

#### 2.2.1. DMA Characterization

Dynamic mechanical analysis (DMA) was performed using a TA Instruments HR20 rheometer in tension mode. Sample strips had dimensions of 40 mm (length), 8 mm (width), and 23 µm (thickness).

To identify the linear viscoelastic region (LVR) of the material, preliminary strain sweep tests were carried out at 40 °C. These tests were conducted at oscillation frequencies of 0.1 Hz, 1 Hz, and 10 Hz, with strain amplitudes ranging from 0.001% to 1%.

Frequency sweep was performed at 40 °C, over a frequency range of 1 to 10 Hz under a constant tensile force of 2 N.

For thermal analysis, a temperature sweep was conducted from 50 °C to 550 °C at a fixed frequency of 1 Hz. The heating rate was set to 3 °C/min, with an oscillation strain of 0.1% and a constant tensile load of 1 N. All temperature-dependent tests were performed under a continuous nitrogen flow to prevent thermal oxidation.

#### 2.2.2. Thermogravimetric Analysis

Thermogravimetric analysis (TGA) was carried out using a TA Instruments Discovery TGA 55. Samples were placed in platinum crucibles and heated from 25 °C to 800 °C under a nitrogen atmosphere (flow rate: 40 mL/min). The heating rate was fixed at 10 °C/min.

#### 2.2.3. SEM Characterization

Scanning electron microscopy (SEM) was used to characterize the morphology of the nanostructure (zinc oxide nanorods) present in the nano-fMEAs. In this work, SEM analysis was conducted using a Zeiss Sigma 300 microscope (Zeiss, Oberkochen, Baden-Württemberg, Germany). Imaging was performed at a working distance of 5 cm with a beam energy of 3 keV.

#### 2.2.4. EIS Characterization

Electrochemical impedance spectroscopy (EIS) was carried out to characterize the electrical properties of the fMEA. EIS was conducted using an AMETEK-VersaSTAT^®^ 4 potentiostat (Ametek Scientific Instruments, Oak Ridge, TN, USA). Measurements were performed in phosphate-buffered saline (PBS, pH 7.4), a widely used electrolyte in biological research due to its ability to mimic physiological conditions (Sigma-Aldrich, St. Louis, MO, USA; catalog number P4417).

A platinum (Pt) electrode served as the reference electrode, while a gold (Au) electrode functioned as the counter electrode. The fabricated ZnO electrodes were used as the working electrodes. Experiments were carried out at room temperature in a Faraday cage, using a DC bias of 0 V and an AC signal of 10 mV over a frequency range of 0.1 Hz to 10 kHz.

### 2.3. System Testing

#### 2.3.1. Biological Sample Preparation and Perfusion

All procedures involving the use of laboratory mice were performed in accordance with the European Communities Council Directive of 24 November 1986 (86/609/EEC) and the Animal Welfare Guidelines approved by the Institute of Experimental Medicine of the Czech Academy of Sciences (approval number 50/2020). Three-month-old male and female *Tg(GFAP-EGFP)1Hket* transgenic mice (*n* = 6) were used for each experiment, providing 18 brain slices for OGD and 18 slices for 10 mM K^+^ stimulation. In this mouse line, the visualization of GFAP-positive cells, such as astrocytes, is feasible due to the expression of enhanced green fluorescent protein (EGFP) under the control of the human glial fibrillary acidic protein (GFAP) promoter. The mice were generated on the FVB/N background strain [39]. Recordings were primarily focused on the cortical and hippocampal regions, as these areas are highly responsive in the context of ischemia [40]. Mice were deeply anaesthetized with sodium-pentobarbital (100 mg/kg, intraperitoneally), transcardially perfused with cold (4 °C) isolation solution, and decapitated. The brains were quickly dissected, and 300 µm thick slices were prepared using a cooled Leica VT1200S vibrating microtome. The slices were kept for 30 min in the isolation solution at 34 °C, followed by another 30 min at room temperature in artificial cerebrospinal fluid (aCSF). The slices were subsequently kept at room temperature in aCSF for a maximum of 5 h. Two experimental solutions were employed to mimic ischemic conditions in acute brain slices: OGD solution or aCSF with elevated potassium concentration (10 mM K^+^), referred to as hyperkalemic solution. In our experimental setup, brain slices were exposed to the stimulus for 5 min. The velocity of perfusion was 4.6 mL/min (peristaltic pump PCD 31.2, Kouril, CZ). The composition of solutions is listed in Table 1. All bicarbonate solutions, except for OGD, were gassed with 95% O_2_ and 5% CO_2_ to maintain a pH of 7.4. The OGD solution was saturated with 5% O_2_, 5% CO_2_, and 90% N_2_.

#### 2.3.2. Cell Culture Preparation for In Vitro Biocompatibility Testing

The neural stem/progenitor cell (NS/PC) culture was prepared as previously described [41]. Briefly, after decapitation, the frontal lobe of the neonatal GFAP/EGFP mouse brain was quickly isolated. Using a 1 mL pipette, the tissue was mechanically dissociated. The cells were subsequently filtered through a 70 µm cell strainer into a Petri dish with proliferation medium containing Neurobasal-A medium (Life Technologies, Waltham, MA, USA), supplemented with B27 supplement (B27; 2%; Life Technologies, Waltham, MA, USA), L-glutamine (2 mM; Sigma-Aldrich, St. Louis, MO, USA), antimicrobial reagent primocin (100 μg/mL; InvivoGen, Toulouse, France), bFGF (10 ng/mL), and EGF (20 ng/mL); both were purchased from PeproTech, Rocky Hill, NJ, United States. The cells were cultured as neurospheres at 37 °C and 5% CO_2_. After 7 days, the neurospheres were collected and centrifuged. The supernatant was discarded, and 1 mL of trypsin (Sigma-Aldrich, St. Louis, MO, USA) was added to the pellet. After 3 min of trypsin incubation, 1 mL of trypsin inhibitor (Sigma-Aldrich, St. Louis, MO, USA) was added to the mix. The suspension was then centrifuged at 1020× *g* for 3 min and plated onto ZnO nanorods and poly-L-lysine (PLL)-coated (Sigma-Aldrich, St. Louis, MO, USA) coverslips (control surface) at a cell density of 6 × 10^4^ per surface. The cells were cultured in a differentiation medium with the same composition as the proliferation medium, but devoid of EGF and with a twofold (20 ng/mL) concentration of bFGF. Cultures were maintained at 37 °C and 5% CO_2_, with medium exchange on every third day. After 7 days of in vitro differentiation, the cells were fixed in 4% paraformaldehyde solution and stained for the astrocytic marker GFAP (mouse anti-GFAP coupled to Alexa 488, 1:300; Ebioscience, San Diego, CA, USA). To visualize cell nuclei; the coverslips were incubated with 300 nM 4′,6-diamidino-2-phenylindole (DAPI; Molecular Probes, Carlsbad, CA, USA) in PBS for 5 min at room temperature. Notably, immunostaining for GFAP at day 7 in vitro does not conflict with the initial GFAP–EGFP signal, since the EGFP synthesized at day 0 undergoes proteasomal degradation (t_1_/_2_ ≈ 6–7 h) and is further diluted by successive mitotic divisions, leaving minimal residual fluorescence after 7 days in culture [42].

Finally, the coverslips were mounted using Aqua Poly/Mount (Polysciences Inc., Eppelheim, Germany).

#### 2.3.3. Recording Setup and Procedure

**Figure 1:**
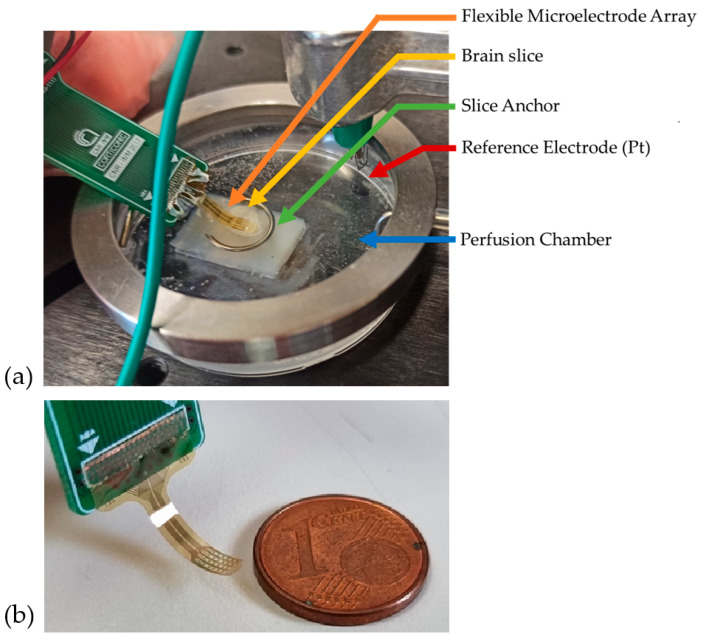
(**a**) Recording setup; (**b**) size comparison of flexible MEA.

The signal acquisition setup is depicted in Figure 1a. Recordings were conducted in a 5 cm plastic perfusion chamber. To facilitate handling, a 0.5 cm agar gel layer was affixed within the chamber, serving as a support for the brain slice. The brain slice was placed on top of the agar, and the MEA was precisely positioned using an electronic micromanipulator-controlled system (Luigs & Neumann, Ratingen, Germany), which enabled fine, controlled movements of the electrode.

Once the fMEA was in contact with the brain slice, it was secured using a non-conductive horseshoe-shaped anchor. A platinum filament served as the reference electrode and was submerged in the aCSF. The use of a platinum electrode instead of an Ag/AgCl reference was primarily dictated by constraints associated with the experimental setup used for signal recording. Nonetheless, since our main focus was on relative changes in potential during spontaneous or evoked neuronal activity, rather than on absolute potential values, the use of a platinum reference electrode did not compromise the validity of our results. To minimize external interference, the entire system was enclosed within a Faraday cage. The small-scale dimensions of the MEA are shown in Figure 1b.

#### 2.3.4. Signal Acquisition and Performance Evaluation

Signal acquisition tests followed a standardized protocol consisting of three phases: a baseline phase in aCSF solution for the detection of spontaneous activity, a stimulation phase in which the slice was subjected to either hyperkalemia or OGD, and a post-stimulation phase in which the slice was perfused with the standard aCSF. Each phase consisted of a 5 min recording, representing a total of 15 min per test.

## 3. Results

### 3.1. Nanostructure Morphology and Characterization

**Figure 2:**
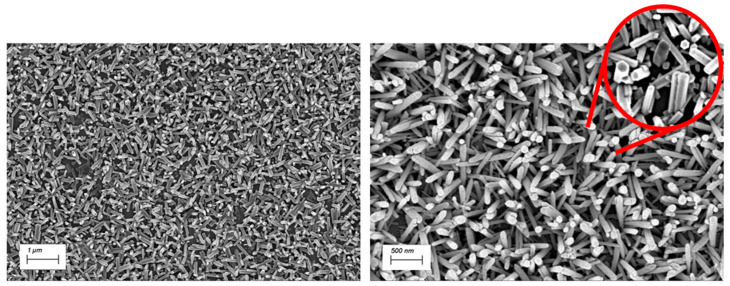
SEM images of zinc oxide nanorods (ZnO NRs) at two different magnifications. The hexagonal structure is notable in the inset of the picture.

The ZnO NRs synthesized via chemical bath deposition form a dense, homogeneous, and randomly oriented nanostructured surface. As shown in Figure 2, the ZnO NRs exhibit a hexagonal wurtzite crystal structure. By restricting the synthesis time to 1 h, the resulting nanorods reach lengths of 600–900 nm, with thicknesses ranging from 100 to 150 nm.

### 3.2. Dynamic Mechanical Analysis

The viscoelastic properties of polymeric substrates intended for neural implants are critical to their functional performance. These materials must endure physiological oscillatory stresses, particularly within the linear viscoelastic region (LVR), to ensure mechanical stability and long-term functionality. This is especially relevant for bioelectronic interfaces implanted in the brain, where mechanical mismatch between the implant and neural tissue can lead to inflammation and signal degradation.

The preliminary strain sweep conducted at 40 °C, a temperature chosen to approximate the internal environment of a rodent brain, identified the LVR of the nano-fMEAs. The material exhibited a linear stress–strain relationship up to a strain amplitude of approximately 0.2%, with the storage modulus (E′) remaining constant throughout this range. This confirms the elastic behavior of the material under small deformations, which is essential for minimizing mechanical damage to surrounding neural tissue during implantation and operation.

**Figure 3:**
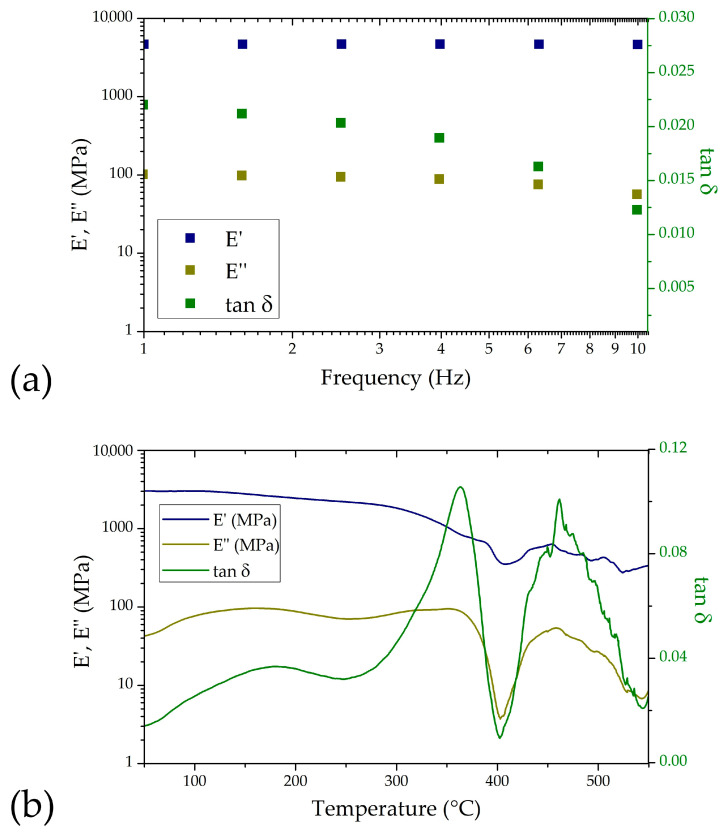
DMA characterization of nano-fMEA: (**a**) frequency sweep, performed at 40 °C; (**b**) temperature sweep, performed at 1 Hz.

To simulate physiological loading conditions, a frequency sweep was performed between 1 and 10 Hz—reflecting the frequency range of cardiovascular pulsations [43] (e.g., heartbeat-induced micromotions) that the implanted device would experience. As shown in Figure 3a, the storage modulus (E′), loss modulus (E″), and damping factor (tan δ) are reported as a function of frequency. As expected, E′ was consistently higher than E″ across the entire range, indicating a predominantly elastic response. The low values of tan δ suggest minimal energy dissipation, which supports the mechanical stability of the device during cyclic loading.

Temperature-dependent DMA analysis further evaluated the thermal–mechanical behavior of the coated polyimide films. As shown in Figure 3b, E′, E″, and tan δ were monitored over a temperature range from 50 to 550 °C. The glass transition temperature (Tg) was identified at around 360 °C from the peak in the loss modulus and tan δ curves. This transition marks the point where the polymer begins to exhibit more rubber-like behavior, and understanding it is crucial for defining the upper operational temperature of the electrode structure. From these analyses, it is evident that the material is very stable and suitable for the intended application.

### 3.3. Thermal Characterization

**Figure 4:**
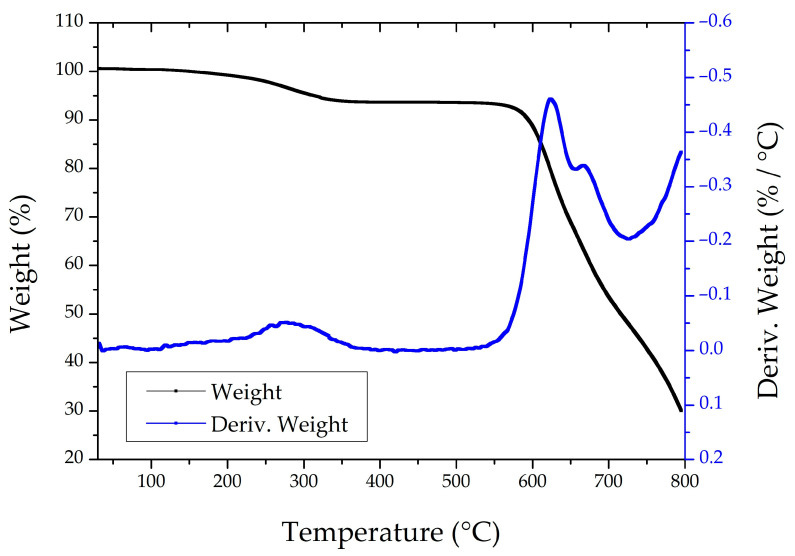
TGA analysis of nano-fMEA under a nitrogen atmosphere. The black curve shows the weight loss as a function of temperature, while the blue curve represents the derivative of the weight loss, indicating the rate of decomposition with respect to temperature.

The results from the TGA are reported in Figure 4. The analysis revealed a three-stage degradation profile for the polyimide-based samples incorporating ZnO nanostructures and metallic coatings. The first notable weight loss occurred around 250 °C, likely due to the degradation of low-molecular-weight species, such as residual solvents, unreacted monomers, or curing agents from the polyimide synthesis. Additionally, organic molecules or surface stabilizers adhered to the ZnO nanostructures may also have contributed to this initial mass loss.

The primary degradation step was observed near 620 °C, corresponding to the thermal decomposition of the polyimide backbone, including chain scission and the breakdown of aromatic and imide groups. A secondary peak around 670 °C may be attributed to the delayed degradation of residual polyimide layers, particularly in encapsulated or metal-adjacent regions where reduced heat transfer slows decomposition.

At 800 °C, approximately 30% of the sample mass remained, attributable to the presence of thermally stable ZnO nanostructures and inorganic residues from titanium and gold coatings, which remain undecomposed under inert conditions.

The temperature-dependent characterizations performed confirmed the thermal stability of ZnO nanorods under conditions relevant for device fabrication and sterilization processes, which could also guide their potential transfer and integration into other biomedical or electronic platforms.

### 3.4. Electrical Characterization

**Figure 5:**
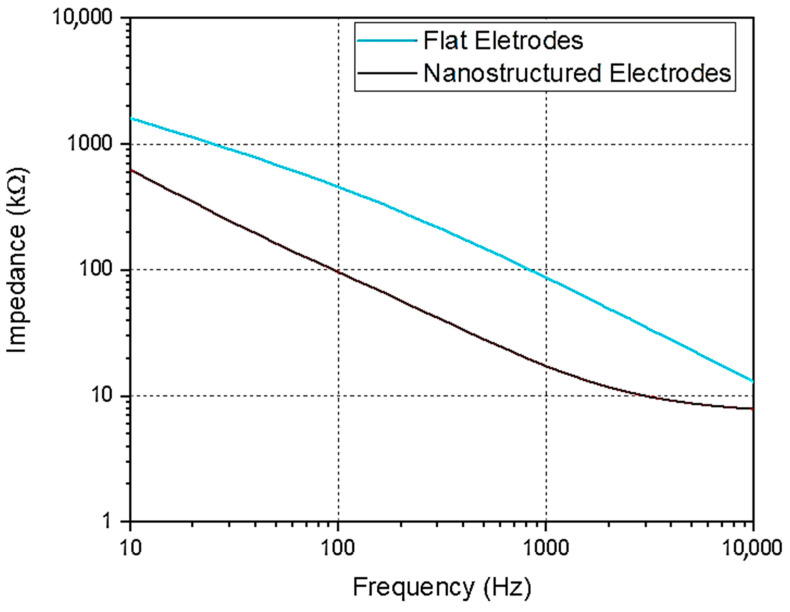
EIS of nanostructured and flat flexible electrodes.

The primary objective of developing nanostructured MEAs is to enhance electrode performance by increasing the electrode’s surface area and therefore reducing impedance values. The EIS measurements were carried out to evaluate electrode performance. The results, shown in Figure 5, demonstrate a significant impedance reduction at low frequencies. At 1 kHz, flat fMEAs exhibited an impedance of 89 ± 3 kΩ, whereas nano-fMEAs demonstrated a reduced impedance of 17 ± 2 kΩ, representing an ~80% improvement.

These findings are aligned with impedance values obtained for other nanostructured electrodes of rigid recording platforms (i.e., ZnO NRs grown on glass substrates [24]), validating that the improvements are also maintained on flexible MEA configurations.

### 3.5. Device Design

**Figure 6:**
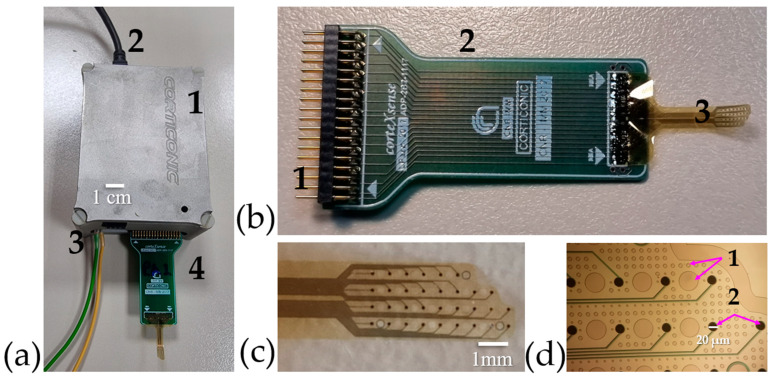
Nano-fMEA and its components. (**a**) Custom-made system developed at CNR-IMM with: (a1) acquisition board, (a2) connection to computer and energy source, (a3) grounding, and (a4) fMEA. (**b**) fMEA with: (b1) pins to interface with the system, (b2) custom-made PCB adaptor, and (b3) polyimide-based grid. (**c**) fMEA highlighting its asymmetric design, which is tailored for recordings on the slice or wrapped around it. (**d**) Zoom-in of the nano-fMEA, which highlights: (d1) the open holes that allow oxygenation and (d2) the nanostructured recording pads, with a size of 20 µm.

The fabrication techniques used result in an ultra-thin fMEA. By using polyimide as both substrate and passivation layer, the resulting MEA is just over 8 µm thick, while maintaining conformability. Details of the complete recording device and its components can be seen in Figure 6a,b. The fMEA presents two key features aimed at enhancing its functionality. Firstly, it features an asymmetric design, as illustrated in Figure 6c, which allows for fabrication in both left- and right-oriented configurations. This design choice enhances the device’s versatility. While this study focuses on the use of fMEA for ex vivo slices, its asymmetric structure has the potential to support in vivo testing by covering an entire hemisphere in most laboratory rodents. In acute brain slice preparations, electrode access through the cut edge of the tissue can influence recording quality and cellular integrity. At the cut edge, neuronal membranes are more exposed and potentially damaged, which may compromise signal stability or distort the local extracellular environment. By contrast, accessing the cortical surface preserves a more intact and physiologically representative architecture, supporting more consistent recordings and reducing variability in spontaneous or evoked activity. Although the asymmetry of the probe design helps adapt to the cortical surface, its use could be further optimized to favor surface access rather than relying on the cut edge, thus improving the relevance and reproducibility of the collected data. Additionally, the design includes open holes, as shown in Figure 6d, which facilitate oxygenation of the slice and improve surface adhesion.

### 3.6. Assessment of Nano-fMEA Biocompatibility in Neural Cells

**Figure 7:**
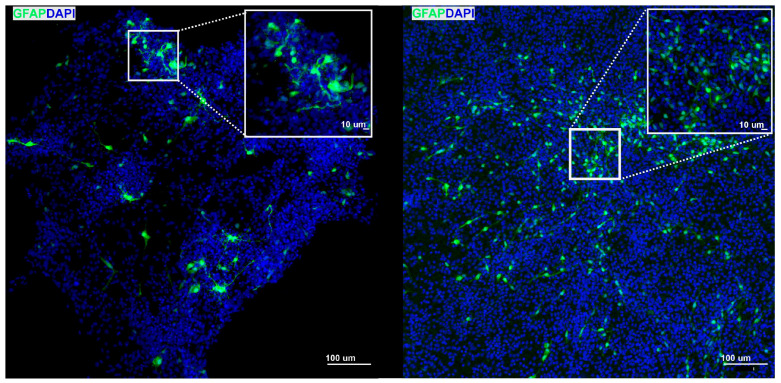
Example of a qualitative acquisition of NS/PC-derived astrocytes grown on control, poly-L-lysine-coated surfaces (**left**) and on ZnO surfaces (**right**) for 7 days in in vitro conditions; astrocytes are visualized in green (GFAP) and cell nuclei are in blue (DAPI). GFAP, glial fibrillary acidic protein; DAPI, 4′,6-diamidino-2-phenylindole.

To assess the biocompatibility of ZnO nanostructures with neural cells, the overall cell fitness and attachment were evaluated by seeding NS/PCs, derived from neonatal GFAP/EGFP mice, onto ZnO substrates. PLL-coated glass coverslips served as the control surface as they represent a widely accepted in vitro standard for optimal cell adhesion. After 7 days of in vitro culture, the samples were fixed and stained for the astrocytic marker GFAP and the nuclear marker DAPI. Although astrocytes on the ZnO nanorods initially appeared smaller and more rounded than those on control surfaces, indicating a modest early attachment challenge, they nonetheless proliferated and achieved a differentiated morphology by day 7 (Figure 7), confirming that the ZnO substrates support neural cell compatibility.

### 3.7. Signal Acquisition with Nano-fMEAs in Acute Brain Slices

While in vitro cultures of neural cells were employed for assessing nano-fMEA biocompatibility at the single-cell level, they lack the complex three-dimensional architecture, synaptic connectivity, and extracellular matrix of intact tissue. To better capture these features, we turned to acute brain slices: ex vivo MEA recordings preserve the native cellular microenvironment and local network connectivity, enabling detection of spontaneous and evoked neuronal activity under near-physiological conditions. However, signal acquisition in acute slices poses additional challenges, such as restricted tissue viability, lower cell density at the electrode interface, and increased impedance, compared with in vivo testing. Therefore, network activity is present as weaker signals of lower amplitude. Weaker signals, in addition to a higher sensitivity of cortical slices to changes in the environment, result from recording conditions that are more sensitive to noise. Therefore, baseline noise was evaluated during preliminary electrode characterization in PBS, pH 7.4, without the presence of biological tissue, for both flat fMEA and nano-fMEAs. Under these conditions, the nano-fMEAs exhibited significantly lower intrinsic noise, with a root-mean-square (RMS) noise level of ~30 µV, compared to ~65 µV for flat fMEAs. These values reflect the differences in electrode impedance between the two geometries and are not directly related to the traces shown in the experimental recordings.

**Figure 8:**
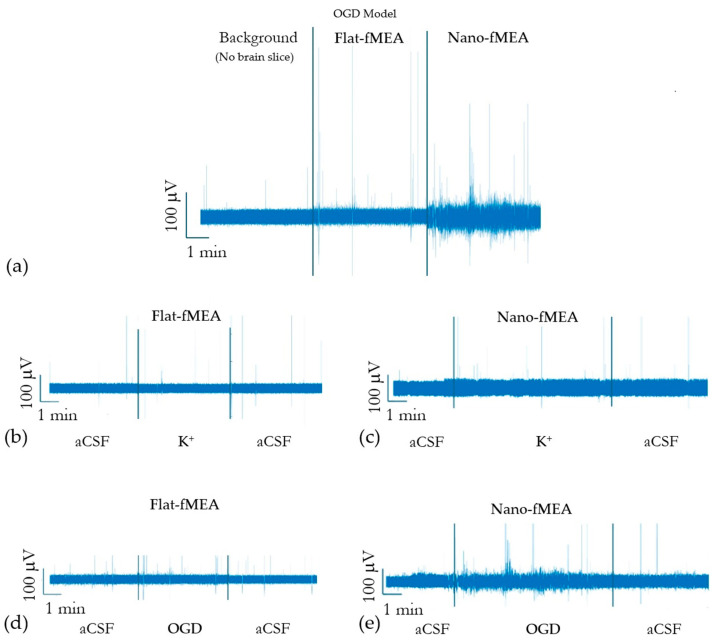
(**a**) Comparison of activity detection between a traditional flat flexible grid (flat fMEA) and the nanostructured flexible MEA (nano-fMEA) compared to the background noise in OGD conditions. Recording of hyperkalemic stimulation using (**b**) flat fMEA and (**c**) nano-fMEA. Recording of OGD model using (**d**) flat fMEA and (**e**) nano-fMEA.

Stimulation with experimental solutions (hyperkalemia and OGD) was used to evaluate the influence of ZnO NRs on the recording capacities of the fMEA. While both fMEA designs detected activity, the nano-fMEA design exhibited significantly improved signal acquisition. Figure 8a showcases the difference in signal detection between the two MEA designs in the OGD model, disclosing increased signal acquisition when recording with nano-fMEA (raw data).

**Figure 9:**
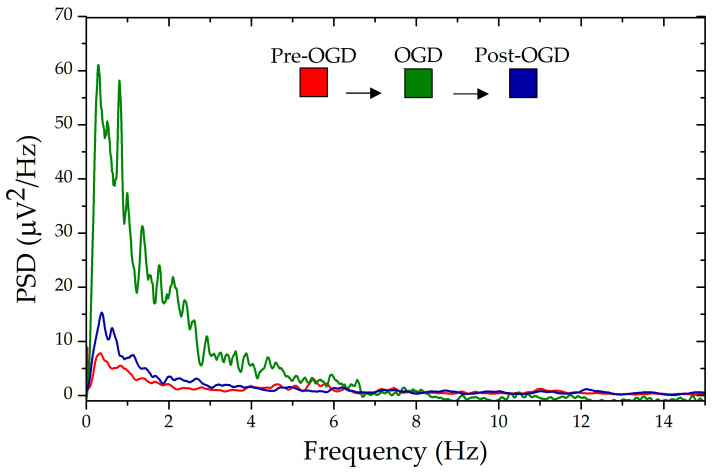
Power spectral density (PSD) obtained with nano-fMEAs during the OGD protocol. As expected, brain activity from the starting levels (red traces) increases due to oxygen and glucose deprivation (green trace), and then it returns to the starting levels (blue trace).

In the hyperkalemic model, where elevated extracellular potassium induces neuronal depolarization and increases spontaneous activity, the nano-fMEA demonstrated a higher event detection rate compared to flat fMEAs, capturing a greater number of spike occurrences with improved signal clarity, as can be seen in (Figure 8b,c). This enhanced sensitivity can be attributed to the optimized electrode–tissue interface, which reduces impedance and improves charge transfer. The advantage of the nano-fMEA became even more pronounced in the OGD model, which simulates ischemic conditions by depriving tissue of oxygen and glucose, leading to metabolic stress and altered neural firing patterns. Under these conditions, the nano-fMEA not only recorded a significantly greater number of events than its flat counterparts but also detected subtle fluctuations in neural activity that were otherwise undetected, as shown in (Figure 8d,e). The increased difference in event detection during OGD suggests that the nano-fMEA is particularly suited for capturing weak or transient signals, likely due to its conformal contact and enhanced electrode properties. These findings highlight the potential of nano-fMEAs for studying pathological neural dynamics with greater precision, especially in ischemic models and metabolic stress. As shown in Figure 9, the power spectral density (PSD) recorded in the three different phases of the OGD protocol clearly highlighted the increase in neural activity during the deprivation period and a similar, reduced activity between the pre-OGD and post-OGD phases. This trend is specifically significant at very low frequencies (<10 Hz), highlighting the ability of this interface to detect slow oscillations in the brain slices, a feature that can rarely be observed with other biointerfaces and is limited to other disordered nanostructures like those with Si NWs [44]. Similar behavior was observed during hyperkalemia, highlighting the superior properties of the nano-fMEA in capturing these signals compared to flat devices.

## 4. Discussion

The fabrication of the nano-fMEAs and the realization of portable electronics for signal recording represent a bioengineering system specifically designed for the investigation of brain activity in vitro and ex vivo. The usage of ZnO disordered nanostructures offers multiple advantages related to the reduction of the impedance of the electrodes (up to 80% lower than flat electrodes at 1 kHz for a fixed area), the low growth temperature (85 °C) and good uniformity of nanostructures (they can be grown on an area up to 28 squares inches) that make the material compatible with ultra-thin flexible substrates, and the good biocompatibility. The nano-fMEA grid has been designed with a high number of holes (up to 50% of the whole area) to facilitate the passage of nutrients and oxygen into the brain tissue during the experiments, to maintain good conditions in vitro and ex vivo before and after the deprivation period, and also to favor the diffusion of the experimental solutions to better mimic ischemic conditions in the whole volume of the brain slice.

In this study, we demonstrated that nano-fMEAs exhibit enhanced detection sensitivity compared to traditional flat MEAs in capturing both rapid and subtle electrophysiological responses during OGD and hyperkalemia.

Nano-fMEA biocompatibility was tested in vitro, visualizing NS/PC-derived GFAP-positive cells on ZnO and control substrates. NS/PCs were chosen for their high proliferative and differentiation capacity, and their adaptability to a range of surfaces, features that make them an ideal model for biocompatibility assays [45]. Among other neural cells, astrocytes were selected as a representative cell type to evaluate ZnO substrates’ biocompatibility. Under optimal conditions, astrocytes develop a characteristic branched morphology; conversely, when growth conditions are suboptimal, they assume a rounded, less differentiated shape [46]. As shown in Figure 7, astrocyte differentiation remained robust: by day 7 in vitro, cells on ZnO nanostructures exhibited physiological, mature branching, comparable to that of cells grown on PLL-coated control surfaces or on other nanostructures such as silicon nanowires [44]. In our experiments, GFAP-based immunostaining was primarily intended to visualize astrocytes. However, it is important to realize that the cultures utilized in our in vitro experiments were prepared from NS/PCs residing in the neurogenic niche of the subventricular zone. This means that our cultures contained, besides GFAP-positive astrocytes, stem cells that also stain for GFAP [47,48] and possess a less elaborate morphology. Nevertheless, the branched morphology of the cells observed after 7 days in culture indicated a suitable surface for cell attachment and no significant cytotoxicity of nano-fMEAs, supporting their suitability for long-term neuroelectronic interfaces. However, this suitability is difficult to validate in in vitro or in situ conditions, as cell cultures and brain slices have a limited lifespan.

Therefore, only acute, short-term ex vivo extracellular recordings on nano-fMEA were performed. In our setup, brain slices were exposed to OGD for 5 min, a duration that has been previously shown to be sufficient to trigger hallmark ischemic responses [49,50], including ATP depletion, glutamate release, and membrane depolarization. Although we did not measure these parameters directly, the time-dependent changes observed in the extracellular traces during OGD were compatible with alterations in ionic dynamics associated with these processes, as reported in the literature [51]. In addition, hyperkalemia was used to induce ionic imbalance and elicit SDs [52]. In the healthy brain, extracellular K^+^ levels typically range between 2.5 and 3.5 mM; during ischemia, this concentration rises dramatically, reaching 10–12 mM in the early phase (within 2 min), and exceeding 80 mM in the later phase [53,54]. Our protocol mimics an early ischemic phase, wherein neuronal recovery is expected upon return to normal conditions. Both OGD and hyperkalemia serve as complementary models to study ischemia-related mechanisms in brain slices, enabling detailed investigation of ion dynamics and cellular responses under ischemic stress in the future.

Overall, our comparative recordings revealed that nano-fMEAs outperform conventional flat MEAs in both models, detecting a greater number of high- and low-amplitude events. It is important to note that acute brain slices provide a valuable model for studying ischemia-induced neural activity, preserving local microcircuitry and enabling precise environmental control and high-resolution recordings; however, they also present intrinsic limitations, including partial neuronal loss, severed afferent and efferent connections, and the absence of modulatory input from distant brain regions [55,56].

As a result, spontaneous activity tends to be sparse and inconsistent in ex vivo preparations [57,58], which likely explains the low frequency of events observed during pre- and post-stimulation phases. Additionally, the choice of recording location within the brain critically influences data quality: neurons located near the cut edge of a slice often exhibit greater injury and disrupted physiology, whereas those near an intact cortical surface typically retain physiological membrane properties and network integration [59]. In this study, we positioned the recording electrodes on the cut edge of the cortical/hippocampal region of 300 µm thick coronal brain sections; however, the sensitivity of the nano-fMEA also allowed signals from viable cells in the deeper layers of the neural tissue (i.e., below the cut surface) to be recorded. Additionally, the asymmetry of the probe design minimized mechanical damage and optimized tissue contact. Therefore, our nano-fMEAs consistently captured more of the pre- and post-stimulation events, underscoring their superior sensitivity when compared to regular flat fMEAs (Figure 8).

Moreover, during the 5 min of OGD stimulation, event frequency rose markedly. Conversely, hyperkalemic stimulation evoked fewer, more transient events. These results are consistent with the different severity of the applied stimuli: hyperkalemia increases neuronal excitability by depolarizing membranes but does not compromise cellular metabolism and generally does not elicit strong glial responses [60,61]. On the contrary, OGD, by depriving tissue of oxygen and glucose, induces broader ionic disturbances, metabolic stress, and glial activation, resulting in a more complex and sustained cascade of responses [62]. Thus, the higher signal activity observed during OGD reflects its stronger and more disruptive effect (Figure 8). Interestingly, OGD stimulation allowed recording of low-amplitude fluctuations in neural activity, likely reflecting attenuated responses from neurons located at a greater distance from the electrode, combined with local glial contributions [63,64]. Under ischemic stress, many neurons depolarize, yet fail to reach spike threshold, generating low-amplitude excitatory and inhibitory postsynaptic potentials that are normally buried in the background noise. Simultaneously, activated astrocytes produce slow, small-voltage field potentials through potassium buffering, gliotransmitter release, and calcium waves [65,66]. The ZnO nanorod coating on the nano-fMEA lowers electrode impedance and increases the slice–electrode contact area, boosting sensitivity, particularly to these low-amplitude, low-frequency events that standard flat MEAs are not able to detect. Finally, PSD analysis also confirmed increased neural responses during OGD and proved that the events are stimulus-driven: spectral power rose significantly during the 5 min stimulation phase and returned to baseline afterwards, ruling out recording artifacts (Figure 9).

Taken together, our findings establish nano-fMEAs as a potential tool to detect the complex interplay of neuronal and glial dynamics under metabolic and excitotoxic stress. Their high sensitivity to subtle, transient events and improved biocompatibility make them ideally suited for studies of ischemia-related pathologies in brain-slice models. Future work will aim to integrate targeted chemical blockers to causally attribute recorded signals to neurons and glial cells, and to extend these platforms toward chronic, in vivo applications.

## 5. Conclusions

In this work, we successfully fabricated, characterized, and evaluated the performance of nano-fMEAs for the detection of ischemia-induced neural activity in acute brain slices. Our findings demonstrate that the incorporation of ZnO nanostructures significantly enhances electrode sensitivity, particularly in reducing impedance and improving signal clarity. These advancements address key limitations of traditional flat fMEAs by increasing sensitivity and maintaining conformability to biological tissues, which is critical for both acute and potential chronic applications.

The nanostructured design of our nano-fMEAs resulted in an 80% reduction in impedance at 1 kHz, compared to flat electrodes, which is consistent with previously reported improvements in nanostructured electrodes used in rigid platforms. This reduction in impedance facilitates more accurate electrophysiological recordings, particularly in challenging ex vivo environments where signal amplitude is lower than in in vivo conditions. Additionally, our design improvements, including asymmetric electrode configurations and oxygenation holes, enhance both adaptability and functionality, ensuring more efficient interaction with brain tissue.

Through ex vivo recordings of ischemia models using both OGD and hyperkalemic stimulation, we validated the capability of nano-fMEAs to detect ischemia-related ionic disturbances with high fidelity. Our results confirm that nano-fMEAs are effective in capturing neuronal and eventually glial responses to pathological conditions, offering a valuable platform for investigating ischemic mechanisms and potential therapeutic interventions.

Overall, this work highlights the potential of nano-fMEAs in advancing neural interface technology. Future research can expand the application of nano-fMEAs from ex vivo to in vivo studies. This new design can pave the way for improved diagnostics and treatment strategies for ischemic brain injury and other neurological disorders.

## Figures and Tables

**Table 1:** Composition of the solutions used.

Compounds	aCSF (mM)	Isolation Solution (mM)	Hyperkalemic (mM)	OGD (mM)
NaCl	122	-	115	122
NMDG	-	110	-	-
KCl	3	2.5	10	3
NaHCO_3_	28	24.5	28	28
Na_2_HPO_4_	1.25	1.25	1.25	1.25
Glucose	10	20	10	-
CaCl_2_	1.5	0.5	1.5	1.5
MgCl_2_	1.3	7	1.3	1.3

NaCl, sodium chloride; NMDG, N-methyl-D-glucamine; KCl, potassium chloride; NaHCO_3_, sodium bicarbonate; Na_2_HPO_4_, disodium hydrogen phosphate; CaCl_2_, calcium chloride; MgCl_2_, magnesium chloride.

## Data Availability

The raw data supporting the conclusions of this article will be made available by the authors on request.

## Abbreviations

The following abbreviations are used in this manuscript:

MEAs	Microelectrode arrays
fMEAs	Flexible MEAs
nano-fMEAs	Nanostructured flexible MEAs
ZnO NRs	Zinc oxide nanorods
SD	Spreading depolarization
OGD	Oxygen–glucose deprivation
PCB	Printed circuit board
DMA	Dynamic mechanical analysis
TGA	Thermogravimetric analysis
SEM	Scanning electron microscopy
EIS	Electrochemical impedance spectroscopy
DAPI	4′,6-diamidino-2-phenylindole
GFAP	Glial fibrillary acidic protein
